# Recovery and sequelae in 523 adults and children with tick-borne encephalitis in Germany

**DOI:** 10.1007/s15010-023-02023-w

**Published:** 2023-04-06

**Authors:** Teresa M. Nygren, Antonia Pilic, Merle M. Böhmer, Christiane Wagner-Wiening, Ole Wichmann, Wiebke Hellenbrand

**Affiliations:** 1 Immunization Unit, Robert Koch Institute, Berlin, Germany; 2 Bavarian Health and Food Safety Authority (LGL), Munich, Germany; 3 Institute of Social Medicine and Health Systems Research, Otto-Von-Guericke-University Magdeburg, Magdeburg, Germany; 4 State Health Office Baden-Wuerttemberg (LGA), Stuttgart, Germany

**Keywords:** Tick-borne encephalitis, Sequelae, Severity, Children, Surveillance, Germany

## Abstract

**Purpose:**

Despite being vaccine-preventable, tick-borne encephalitis (TBE) continues to cause considerable morbidity in Germany. Limited insight into potentially debilitating consequences of TBE may partially underly low (~ 20%) TBE vaccine uptake. We aimed to systematically assess TBE sequelae and other consequences.

**Methods:**

Routinely notified TBE patients from 2018 to 2020 from Southern Germany were invited to telephone interviews acutely and again after 18 months. Duration of acute symptoms was prospectively assessed. Recovery was defined as score 0 on the modified RANKIN scale. Determinants of time to recovery were analysed with cox regression, adjusted for covariates identified using directed acyclic graphs, yielding hazard ratios (HR) and 95% confidence intervals (CI).

**Results:**

Of 558 cases, 523 (93.7%) completed follow-up. Full recovery was reported by 67.3% (children: 94.9%, adults: 63.8%). Sequelae included fatigue (17.0%), weakness (13.4%), concentration deficit (13.0%), and impaired balance (12.0%). Compared with 18–39-year-olds, recovery rates were 44% lower in ≥ 50-year-olds (HR: 0.56, 95%CI 0.42–0.75) and 79% higher in children (HR: 1.79, 95%CI 1.25–2.56). The recovery rate was 64% lower after severe TBE (compared to mild; HR: 0.36, 95%CI 0.25–0.52) and 22% lower with comorbidities (HR: 0.78, 95%CI 0.62–0.99). Substantial health-care use was reported (90.1% hospitalisation, 39.8% rehabilitation). Of employed cases, 88.4% required sick leave; 10.3% planned/reported premature retirement due to sequelae.

**Conclusion:**

Half the adult and 5% of paediatric patients reported persisting sequelae after 18 months. Improved prevention could alleviate both individual (morbidity) and societal TBE burden (health-care costs, productivity losses). Insights into sequelae can help guide at-risk populations towards tick-avoidant strategies and encourage TBE vaccination.

**Supplementary Information:**

The online version contains supplementary material available at 10.1007/s15010-023-02023-w.

## Introduction

Rising incidences of tick-borne encephalitis (TBE) in recent years have augmented awareness for this vaccine-preventable disease. The TBE virus is mainly transmitted via *Ixodes ricinus* tick bites, but transmission through unpasteurised dairy products is also possible. While 70–95% of infections with the TBE virus are subclinical [1, 2], an average of 2500 symptomatic TBE cases were notified in Europe every year from 2012 to 2016, of whom 95% were hospitalised [3]. The acute disease phase is relatively well described, typically featuring a biphasic course and symptoms of the central nervous system (CNS) such as meningitis, encephalitis or myelitis [4]. However, less research is available on longer-term sequelae and often only includes adult patients [5, 6].

TBE has been statutorily notifiable to the German infectious disease surveillance system since 2001. Despite the availability of free-of-charge TBE vaccination, vaccination coverage (≥ 3 doses and on-time vaccination) remains stagnantly low around 20% even in high-risk areas in Germany [7]. A median of 529 cases per year was notified in Germany from 2017 to 2020, and around 85% of these originated from the southern federal states Baden-Wuerttemberg and Bavaria [7]. Surveillance data are limited to the acute disease phase [4] and, thus, do not confer insight into recovery or sequelae.

A handful of studies have investigated factors associated with acute TBE severity, but factors underlying sequelae remain understudied. Associations with sequelae were suggested for age [8], severe acute disease [9–11], specific acute symptoms of ataxia or pareses [11], and high acute CSF protein [5, 11]. Several of these studies are, however, limited by the retrospective nature of data collection, often occurring many years after symptom onset, which on the one hand might lead to selection of patients with particularly severe sequelae into the study population and, on the other hand, to inaccuracies due to recall bias. In addition, most studies did not assess sequelae in paediatric patients.

We conducted intensified surveillance of adult and paediatric TBE cases from 2018 to 2020 in Southern Germany. The first data collection point of this prospective cohort study addressed knowledge gaps regarding acute TBE severity, risk factors, places of TBE virus infection, vaccination barriers and vaccine effectiveness, which were published previously [12–15]. We here report data from the second and final data collection point, aiming to systematically characterise and quantify TBE sequelae among notified TBE patients over a period of 18 months after symptom onset.

Besides sequelae, consequences of TBE disease such as health service utilisation, need for rehabilitation, out-of-pocket expenditures, sick leave, consequences on work/school performance, and further harder-to-measure disease outcomes have so far only been studied in limited detail. Recently, several health economic outcomes of TBE were determined in a Swedish register study [16]. To obtain data for the German setting, study interviews also collected details on these outcomes, which are linked to direct and indirect costs of TBE and are, thus, an important input to health economic evaluations.

By comprehensively characterising TBE sequelae alongside wider consequences on patients’ lives, this work aims to raise awareness of the possible severity of TBE virus infection and, thus, contributes to the public health goal of more effective prevention.

## Methods

### Study population and data collection

We invited persons notified with TBE from 2018 to 2020 in Baden-Wuerttemberg or Bavaria to participate. Following written informed consent, initial data collection of this cohort study comprised standardised telephone interviews (30 min) as soon as participants were willing and able to perform these, i.e. on average 3 months after symptom onset. Participants who completed initial interviews were eligible for follow-up, consisting of another interview 18 months after onset. While the report on the acute disease stage, published elsewhere [12], also considered hospital discharge summaries and medical questionnaires, the present analysis focuses on patient-reported outcomes at follow-up. However, comorbidities before TBE onset and health service utilisation collected at the first interview were of course based on/validated with the mentioned medical data sources.

Follow-up interviews covered persistence in months for all symptoms reported at the first interview, except symptoms that only occur acutely (fever, gastrointestinal symptoms, neck stiffness, light sensitivity). Additional symptoms were also recorded. A small number of patients who reported symptoms at initial interview stated not having had a particular symptom at follow-up. Here, the symptom was considered absent. Data on health-care utilisation (e.g. length of hospitalisation) was collected at both interviews. Out-of-pocket non-reimbursed expenditures due to TBE (in €), e.g. for required care services that were not covered by health insurance, were collected retrospectively at follow-up.

### Data analysis

Data management and analysis were performed in R and Stata 17®. We computed descriptive statistics (means, medians, standard deviations (SD), interquartile ranges (IQR), percentages) and used Chi-squared (Chi2) tests and Mann–Whitney U (MWU) to test differences. *P*-values below 0.05 were deemed statistically significant.

Post-encephalitis syndrome (PES) in mild form was defined as the presence of 2–3 subjective symptoms (for symptoms, see Fig. 2) reported as due to TBE virus infection at ≥ 6 months after onset, following the definition in [5]. Since data were self-reported and thus with a risk towards over-reporting of symptoms, we chose more conservative cut-offs than used in [5], with 4–5 subjective symptoms for moderate and ≥ 6 subjective symptoms for severe PES.

For time-to-event analysis, the outcome ‘recovery’ was defined as a score of 0 on the modified RANKIN scale [17], see Supplementary Appendix 1. First, the underlying causal structure was explored with directed acyclic graphs (DAGs) [18] to select the corresponding minimal adjustment set of variables required to estimate the total causal effect of each exposure of interest on recovery (Supplementary Appendix 2). Covariates were tested for equality of survivor functions with log-rank tests. The proportionality assumption was assessed by visually inspecting scaled Schoenfeld residuals. No violation of assumptions was observed. As visual inspection of survival curves indicated non-linear relationships between age and recovery, age was categorised into four strata. Kaplan–Meier curves were obtained and Cox regression was performed, yielding hazard ratios (HR) with 95% confidence intervals (CI).

### Ethics

Cases provided written informed consent. The study protocol was approved by the Ethics Committee of Charité – Universitätsmedizin Berlin, No. EA2/059/18.

## Results

### Study population

**Fig. 1:**
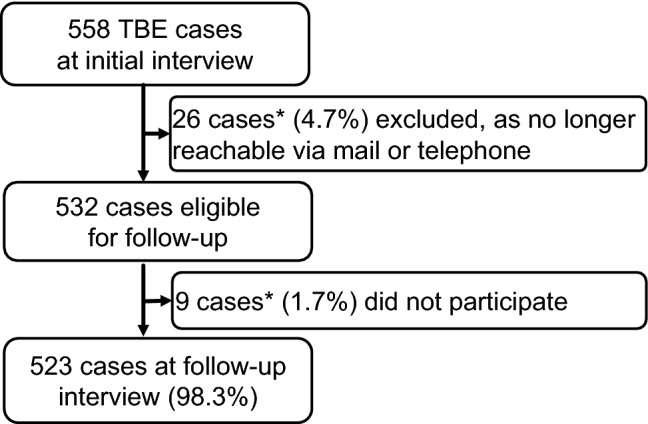
Flowchart of participation and completion of study components. *The 35 patients who did not complete follow-up did not differ from the 523 followed-up patients regarding sex (*p*_Chi2_ = 0.662), age (*p*_MWU_ = 0.403), comorbidities (*p*_Chi2_ = 0.496), RANKIN-score at first interview (*p*_Chi2_ = 0.976), or education ((*p*_Chi2_ = 0.462). Acute TBE severity was slightly milder in these 35 patients (37% mild, 46% moderate, 17% severe) than in patients who completed follow-up (18% mild, 61% moderate, 21% severe, *p*_Chi2_ = 0.025)

Compliance with follow-up data collection was very high, with nearly all eligible cases completing follow-up (98.3%, Fig. 1). The overall follow-up participation rate was 93.7% (523 of 558 cases).

Case characteristics are displayed in Table 1. The majority of cases were male and older than 40 years. Nearly half (43.0%) had comorbidities before TBE onset, most commonly comorbidities of the CNS, hypertension, and chronic-inflammatory comorbidities. A further 54 cases (10.3%) reported additional comorbidities diagnosed before the follow-up, of whom 22 had previously not reported comorbidities. The most common new diagnoses were hypertension (*n* = 19) and diabetes (*n* = 4).

**Table 1:** Characteristics of 523 TBE cases participating in the follow-up interview

Demographics		*n*	%
	Male	333	63.7
	Age group: 2–17 years	59	11.3
	Age group: 18–39 years	80	15.3
	Age group: 40–49 years	80	15.3
	Age group: ≥ 50 years	304	58.1
	Mean age (in years, SD)	48.7	19.9
	Comorbidities at onset	225	43.0
Clinical characteristics during acute TBE			
	Acute severity*: Mild	96	18.4
	Acute severity*: Moderate	320	61.2
	Acute severity*: Severe	107	20.5
RANKIN-score at first interview (~ 3 months after symptom onset)			
	0: no symptoms	255	48.8
	1: no significant disability	159	30.4
	2: slight disability	72	13.8
	≥ 3: moderate disability, or worse	36	6.9
RANKIN-score at follow-up interview (18 months after symptom onset)			
	0: no symptoms	352	67.3
	1: no significant disability	108	20.7
	2: slight disability	42	8.0
	≥ 3: moderate disability, or worse	21	4.0

*For definition of severity, see Supplementary Appendix 6. E.g. severe cases presented with neurological symptoms and were hospitalised for ≥ 3 weeks, or admitted to intensive care, or had myelitis or radiculitis. For details on the RANKIN-score, see Supplementary Appendix 1

For 160 cases (30.6%), follow-up interviews occurred before 22.03.2020, i.e., the start of contact restrictions due to the COVID-19 pandemic in Germany), and for 363 cases (69.4%) after that date, up to 28.04.2022. The interview did not explicitly ask for COVID-19 infection, but 3 patients (0.6%) reported having had COVID-19 when asked for newly diagnosed comorbidities.

Half the cases (48.8%) had recovered by the first interview, which was conducted a median of 93 days (IQR = 66–146 days) after onset. At follow-up, 67.3% reported complete recovery, the interview occurring a median of 552 days after symptom onset (= 18.1 months; IQR = 539–575 days). Full recovery at follow-up was reported by 85.4% of cases with mild acute TBE, 69.4% with moderate, and 44.9% with severe acute TBE (*p*_Chi2_ < 0.001). Full recovery did not differ by sex (males: 68.2%, females 65.8%, *p*_Chi2_ = 0.577), but was more frequent among children (94.9%) than adults (63.8%, *p*_Chi2_ < 0.001). All children aged 2–5 years recovered (*n* = 12), 93% of children aged 6–12 (*n* = 30), and 94% of children aged 13–17 (*n* = 17). Recovery was less common among patients with comorbidities (56.9%) than among those without (75.2%, *p*_Chi2_ < 0.001). For further median times to recovery, see Supplementary Appendix 5.

### Duration of TBE sequelae

**Fig. 2:**
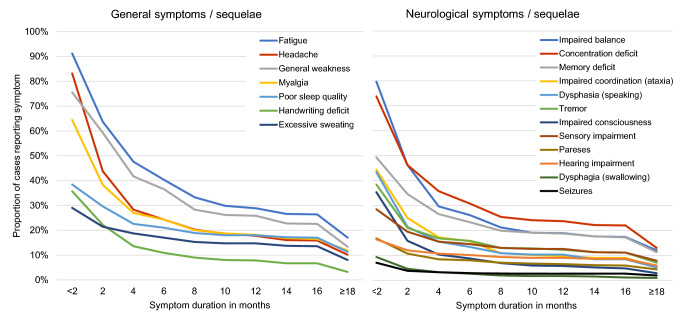
Duration of self-reported TBE symptoms and sequelae (*n* = 523 cases). Detailed percentages are provided in Supplementary Appendix 3. The presence of symptoms was assessed acutely (median: 3 months after onset). Patients provided the duration of then persistent symptoms when interviewed 18 months after illness onset. Affected body parts among cases reporting specific myalgia, tremor, or pareses: Myalgia (reported by 337/523 patients): legs (61.1%), arms (51.0%), back (47.5%), neck (16.6%), shoulders (10.9%); Tremor (reported by 201/523 patients): hands (73.6%), arms (38.3), legs (23.4%), head/neck (9.0%); Paresis (reported by 88/523 patients): arms (64.8%), legs (56.8%), face (38.6%), neck (35.2%), shoulders (35.2%), eyes (25.0%). Among cases reporting hearing impairment (*n* = 86), 62.8% reported sensitivity to sound, 51.2% hearing loss, and 41.9% tinnitus

The most frequently reported acute symptoms reported at the first data collection point were fatigue (91%), headache (83%), impaired balance (80%), general weakness (76%), and concentration deficit (74%). For most cases, symptoms resolved during the observation interval (Fig. 2). The most frequent sequelae reported at 18 months were fatigue (17%), concentration deficit (13%), general weakness (13%), myalgia (12%), and impaired balance (12%). Detailed percentages are provided in Supplementary Appendix 3.

Mild post-encephalitic syndrome (PES) was present in 16% of cases at 6 months, in 12% at 12 months, and in 7% at 18 months. Moderate PES was present in 9%, 10%, and 6% at the respective time points; severe PES in 27%, 20%, and 11%. As displayed in Supplementary Appendix 4, the proportion reporting PES differed by age, with adults reporting PES 3–10 times more frequently than children in any severity form and at any time point.

### Factors determining recovery from TBE

**Fig. 3:**
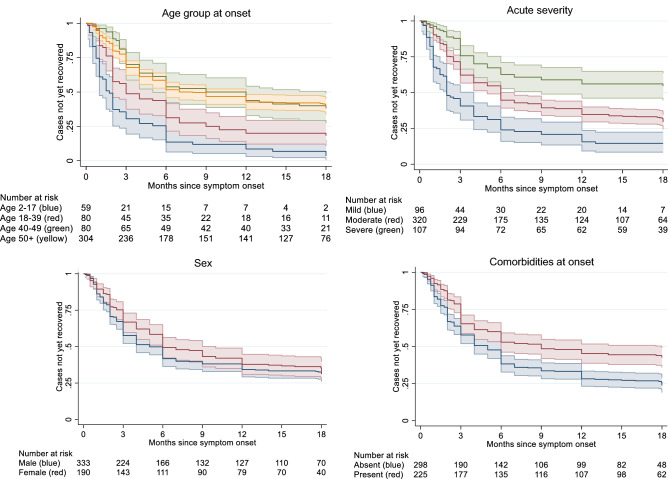
Kaplan–Meier curves on the relationship between complete recovery from TBE symptoms and patient age, acute severity, sex, and comorbidities (*n* = 523)

Kaplan–Meier curves suggest varying recovery rates by age, acute TBE severity and comorbidities (Fig. 3). As described in the methods, age groups were chosen following natural data breaks. Remarkably, recovery rates were nearly identical for each 10-year age stratum between 50 and 90 years, hence these ages were grouped. Recovery at 40–49 years was also similar to recovery at 50–90 years (Fig. 3). Men seemed to recover slightly faster than women, although this difference diminished during follow-up time.

**Fig. 4:**
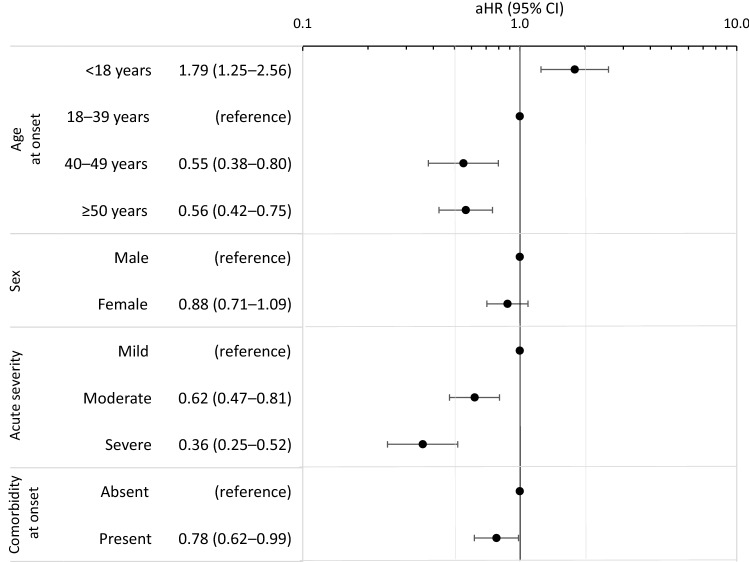
Factors associated with recovery from TBE up to 18 months after symptom onset: Results of multivariable Cox regression analyses (*n* = 523). aHR = adjusted hazard ratio; CI = confidence interval. For univariate estimates, see Supplementary Appendix 5. aHR < 1 equate to longer mean time to recovery, aHR > 1 equate to shorter mean time to recovery. Minimal adjustment sets, as determined with directed acyclic graphs (see Supplementary Appendix 2), for each exposure of interest: Age at onset: No adjustment needed; Sex: No adjustment needed; Acute severity: Age at onset, Comorbidity at onset, TBE vaccination; Comorbidity at onset: Age at onset, Sex, Education. Akaike Information Criterion (AIC) for each model: AIC(Age at onset) = 4057.78, AIC(Sex) = 4108.09, AIC(Acute severity) = 4026.86, AIC(Comorbidity at onset) = 4058.19

Multivariable, adjusted cox regression analysis revealed significant associations between recovery and both age at onset and acute TBE severity (Fig. 4). Compared to cases aged 18–39 years, the recovery rate was 45% and 44% lower for cases aged 40–49 years and ≥ 50 years, respectively. The recovery rate for cases aged < 18 years was 79% higher. Compared to cases with mild acute TBE, the recovery rate was 38% lower after moderate acute TBE and 64% lower after severe acute TBE.

Recovery was not statistically significantly associated with sex (Fig. 4). Comorbidities were associated with 22% lower recovery rate. Given the particular relevance of hypertension for acute TBE severity [12], the analysis was repeated for the comorbidity hypertension alone. The resulting HR for recovery (adjusted for age, sex, education and comorbidities other than hypertension) was 0.85 (95% CI 0.60–1.21), suggesting no particular relevance of hypertension for TBE recovery.

### Health care utilisation and further consequences of TBE

Patients reported substantial health-care utilisation, ranging from intensive care to physiotherapy (Table 2). Seventeen cases (3.3%) were granted assistance through the statutory long-term care insurance (“Pflegegrad”). Data on the type of rehabilitation were available for a subset of 77 cases, of 208 cases receiving rehabilitation. Cases with available data did not differ from cases without data in acute severity (*p*_Chi2_ = 0.496), sex (*p*_Chi2_ = 0.374), or age group (*p*_Chi2_ = 0.072). Of these cases, 88.3% reported receiving physiotherapy, 73.7% received occupational therapy, 36.6% speech therapy, and 58.4% other treatments such as memory training or psychological support. Rehabilitation was rated as very helpful or helpful for alleviating TBE symptoms by 75.0% of the 77 cases with available data. An additional 13.2% were uncertain and 11.8% did not rate the rehabilitative treatment(s) as helpful.

**Table 2:** Health care utilisation and sick leave in 523 TBE patients during the 18 months since symptom onset

Health service/Sick leave	Cases reporting service	Median duratio/number	Unit	IQR	Range
Hospitalisation	90.1%	10	Days	7–15	1–90
Intensive care	12.4%	3	Days	2–6.5	1–62
Mechanical ventilation/intubation	2.1%	5	Days	4–8	2–32
Rehabilitation^a^	39.8%	4	Weeks	3–9	1–80
GP visits	90.2%	5	Visits	3–10	1–180
Additional physiotherapy^b^	31.5%	12	Weeks	6–30	1–76
Additional speech therapy^b^	3.3%	12	Weeks	3–24	1–76
Additional occupational therapy^b^	13.8%	20	Weeks	6–36	1–76
Sick leave from work^c,e^	88.4%	37	Days	21–74	2–595
Sick leave from school^d,e^	68.3%	13	Days	5–23	1–435

^a^Inpatient and outpatient rehabilitation combined; ^b^services in addition to rehabilitation; ^c^denominator: employed cases; ^d^denominator: cases in school; ^e^days hospitalised are excluded, i.e., sick leave days on top of days hospitalised; *IQR *interquartile range, *GP *general practitioner

Although most health services were covered by health insurance, numerous cases reported out-of-pocket expenses. Of the 182 participants who received additional services outside of official rehabilitation such as physio-/occupational or speech therapy (see Table 2), 52.2% reported out-of-pocket expenses for these services at a median of 200€ since TBE onset (IQR = 98–500€, maximum 5000€). Another 24 patients reported out-of-pocket expenses for other care and supporting services, spending a median of 450€ (IQR = 148–1300€, maximum 5000€). Twenty-two cases reported out-of-pocket expenses for supportive medical devices such as walking aids or for adapting their home due to their sequelae, spending a median of 200€ (IQR = 50–1075€, maximum 8000€).

Substantial indirect societal costs arose due to TBE disease or sequelae. Most employed cases required extensive sick leave (Table 2) on top of the days spent in hospital. Four patients (1.2% of employed cases) retired prematurely and at the follow-up interview 31 (9.1% of employed cases) stated that they planned retiring prematurely due to sequelae. Moreover, 104 cases (30.5%) reported negative impacts of TBE on work performance. Of these 104, 41.4% reduced their working hours, 22.1% had to change their position or employer, and 48.1% reported other limitations including cognitive problems (memory, concentration, language), increased fatigue, and deterioration in fine motor skills.

Ten of 43 schoolchildren (23.3%) reported negative impacts of TBE on school performance. Four received worse grades and one pupil had to repeat a schoolyear.

## Discussion

The present study comprehensively characterised TBE symptoms in 523 cases and assessed TBE sequelae up to 18 months after symptom onset. Key findings include differential rates of recovery for symptoms, determinants of recovery rate, and notable societal costs caused by TBE.

Recovery continued throughout the entire observation time, but was fastest during the first 2–4 months after symptom onset. While 49% of patients had fully recovered around 3 months after symptom onset, 67% had fully recovered by 18 months. Symptom resolution rates slowed continually, with only minor improvements reported after around 8 months. Resolution rates differed between symptoms, with quicker resolution of, e.g. headaches, impaired balance, and myalgia, and slower resolution of, e.g. memory deficits, sensory impairment, or hearing impairments. The lower recovery rate after around 8 months seems plausible in light of another publication showing that the proportion of cases with post-encephalitic syndrome was similar at 12 months and at 2–7 years after TBE [5]. This suggests that most patients in our sample reporting sequelae at 18 months are unlikely to recover fully.

The observed proportion of cases with sequelae agrees with previous research. In a Swedish study, 54% of patients had fully recovered after a median of 13 weeks [8]; in a German study 77% of patients only had mild or no sequelae after a median of 12 months [11]. The nature of sequelae is mostly concordant with the literature, with sequelae predominantly in cognitive function, balance, ataxia, and fatigue [5, 6, 8, 11, 19]. One clinically-focussed study highlighted severe neurological sequelae such as paresis or respiratory insufficiency and impaired consciousness [11], however these sequelae only affected few patients in our sample, e. g. 4% with paresis by 18 months. Fatigue, the most common sequela observed here, was not always quantitatively captured in previous studies, or paraphrased as ‘decreased vitality’ [8]. As a recent review pointed out, sequelae of the sleep–wake cycle are common in TBE, manifesting as e. g. fatigue, poor sleep quality, or somnolence [20], with debilitating consequences on daily functioning.

Recovery depended on age, with cases older than 40 years recovering at a 45% lower rate than cases aged 18–39. Notably, recovery rates were nearly identical for patients between 40 and 90 years. Findings are congruent with age being a predictor of both acute TBE severity [12, 21–23] and sequelae [8, 19]. Children had a 79% higher recovery rate, compared to 18–39-age-olds, and more rarely suffered from post-encephalitic syndrome than adults.

The link between acute severity and lasting sequelae corresponds with previous findings [9–11]. Although recovery was possible for acutely severely ill patients (45% did recover fully within 18 months), it occurred at a lower rate. Sex did not affect recovery in our data or elsewhere [8, 19]. The proportion of cases with comorbidities (43%) compares with other studies (e.g. 45% with comorbidities in [5]). In the only other available study investigating the potential link between comorbidities and TBE sequelae, no association was found [5]. Hypertension has been observed to double the odds of severe acute TBE, while chronic-inflammatory comorbidities and those of the CNS slightly increased the odds of severe TBE [12]. Here, the adjusted recovery rate for comorbid patients was 22% lower (95%CI: 1–38%) than for patients without comorbidities, calling for re-investigation in future studies.

The observed health services utilisation was comparable to previous reports, recording a median length of hospital stay of 10 days as in [8, 11, 16] and a comparable proportion of cases receiving rehabilitation as in a German study from 1999 [11]. Sick leave duration was similar to that reported in a Swedish study [16]. The high observed level of additional services beyond rehabilitation, such as physiotherapy or medical devices, often meant significant out-of-pocket expenses. Our detailed data revealed negative impacts on employment capacity and perceived poorer school performance emphasises that TBE and its sequelae can have wide-reaching and long-lasting consequences on patients’ lives.

The main limitation is the self-reported nature of most data. While comorbidities at onset were validated using medical sources, thus minimising the risk of over-reporting or misclassification, follow-up data were not validated. A possible consequence is symptom over-reporting. As the observed sequelae resemble results from clinically-based research (as discussed), over-reporting does however not appear a major concern. Moreover, the recall of sequelae duration may be less reliable for sequelae that resolved in the middle of the follow-up period compared to those resolving during the initial acute phase or during the end of the follow-up period. Risk of misclassification is highest for symptoms that are not easily classifiable by laypersons. Therefore, we avoided medical jargon in patient interviews, e. g. describing tremors as “trembling of the muscles”, but misclassification cannot be entirely excluded. Another limitation is that sequelae were not compared to the background rates of these health deficits in the population. Although interviews specifically asked for TBE-related sequelae, over-reporting is possible for items like concentration deficits, which are prevalent in the general population. A further potential limitation is defining recovery according to the RANKIN scale. Other groups frequently based recovery on medically assessed symptoms (e.g. [8, 11]). While our assessment may be more subjective, it nevertheless has the benefit of capturing a patient-centred outcome that may better translate to daily functioning.

Strengths first include the timely assessment of acute symptoms, along with the prospective assessment of sequelae duration at follow-up. This design minimises the risk of only recalling salient sequelae. Given that follow-up interviews elicited data retrospectively covering an average of 15 months that elapsed since the acute interview, some inaccuracies, e.g. regarding the exact sequelae duration can, however, not be excluded. A second strength is the 94% high participation rate, contrasting some previous studies’ rates at 35–49% [5, 11]. This high rate indicates minimal risk of differential loss-to-follow-up and, thus, distortion of results. Patients who did not complete follow-up were similar to patients completing follow-up in most parameters. The higher proportion of mild acute TBE among the small number of non-participants might, however, have produced a slight over-estimation of sequelae. Third, by capturing manifold consequences of TBE on patients’ lives including employability and out-of-pocket expenses, our work represents one of the most comprehensive accounts to date on this potentially debilitating disease.

## Conclusion

This follow-up study of a large TBE patient cohort up to 18 months after symptom onset revealed details on the persistence of a wide range of general and neurological symptoms. Two thirds of patients recovered fully, especially younger patients or those with less severe acute TBE. However, 36% of adults and 5% of children had not yet fully recovered by 18 months. Of those with severe acute TBE, 55% had not yet recovered. The high and sometimes long-lasting level of morbidity and health-care utilisation underlines the urgent need for improved TBE prevention. Together with recent insights into behavioural TBE risk factors [13] and TBE-specific vaccination barriers [15], our results should be used to inform at-risk population groups on TBE risk and severity, tick-protective strategies and TBE vaccination.

### Supplementary Information

Below is the link to the electronic supplementary material.

Supplementary file1 (DOCX 368 KB)

## Data Availability

The data presented in this article are available on reasonable request from the corresponding author. The data are not publicly available due to ethical and data privacy protection obligations.
