# Supplementary material for: Recovery and sequelae in 523 adults and children with tick-borne encephalitis in Germany
Source: Infection. 2023 Apr 6;51(5):1503–11. doi: 10.1007/s15010-023-02023-w (PMC10078068; doi:10.1007/s15010-023-02023-w)
Supplement: Supplementary file 1 — Supplementary file1 (DOCX 368 KB) [file 15010_2023_2023_MOESM1_ESM.docx]

**Supplementary Information**

# Recovery and Sequelae in 523 Adults and Children with Tick-Borne Encephalitis in Germany

Journal: Infection

Authors: Teresa M. Nygren, Antonia Pilic, Merle M. Böhmer, Christiane Wagner-Wiening, Ole Wichmann, Wiebke Hellenbrand

Corresponding author: Teresa M. Nygren, Affiliation: Immunization Unit, Robert Koch Institute, Berlin, Germany, E-Mail: [NygrenT@rki.de](mailto:NygrenT@rki.de)

**Appendix 1**. Modified RANKIN scale translated to German, as used in study interviews with TBE cases. Full recovery was defined as score 0 on this scale. Source of English original modified RANKIN scale: (1)

Version for adults:


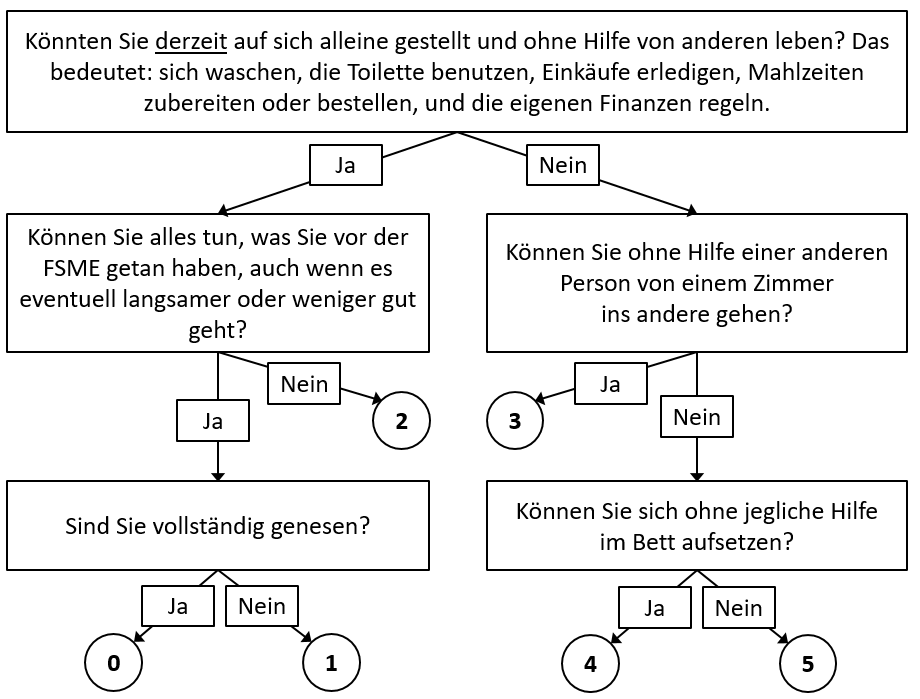


Version for children:


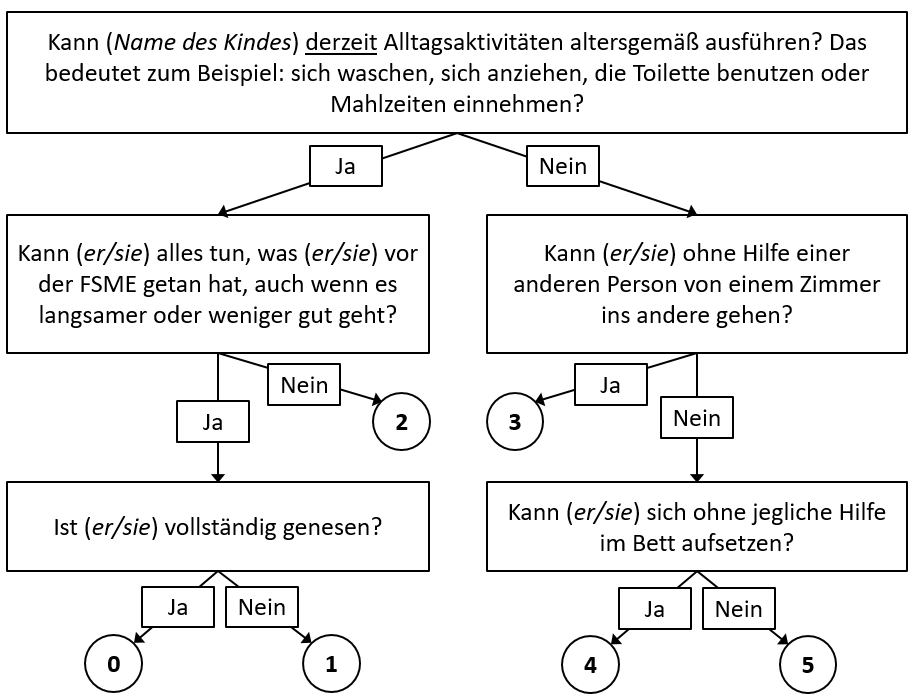


**Appendix 2.** Directed acyclic graph (DAG) of the causal structure underlying mechanisms connecting potential exposures to the outcome recovery from TBE, as determined by subject-specific knowledge. Created with Dagitty (2).


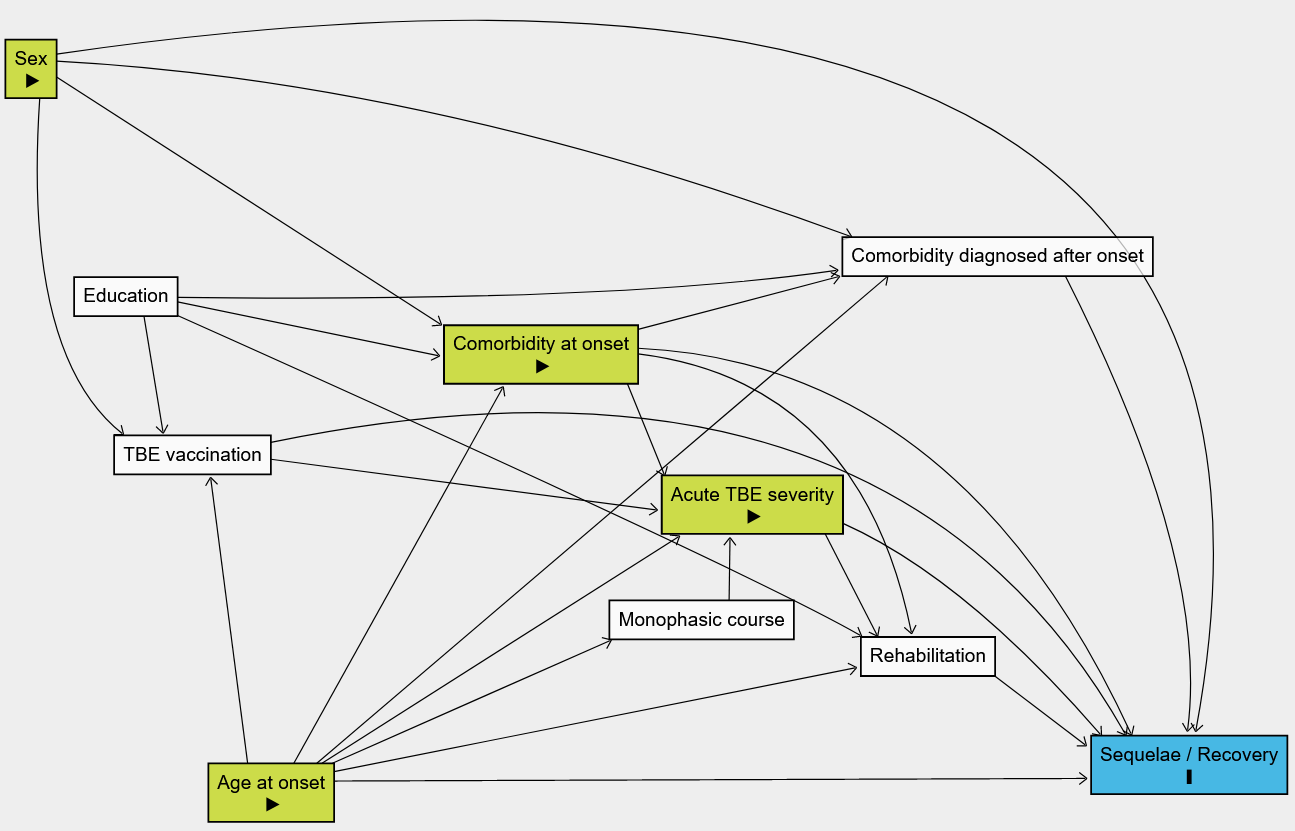


Blue node (**I**) = outcome. Green nodes (►) = exposures of interest. One exposure was selected at a time to identify the minimal sufficient adjustment set of covariates necessary to estimate the adjusted total causal effect of that exposure on the outcome.

**Appendix 3.** Percentage of patients reporting TBE symptoms at different time points

|  |  | Symptom | Proportion of cases reporting symptoms at different time points (in months) since symptom onset | | | | | | | | | | | Data | |
| --- | --- | --- | --- | --- | --- | --- | --- | --- | --- | --- | --- | --- | --- | --- | --- |
| General symptoms/sequelae | | absent | <2 | 2 | 4 | 6 | 8 | 10 | 12 | 14 | 16 | ≥18 | missing | |  |
|  | Fatigue | 8% | 91% | 64% | 48% | 40% | 33% | 30% | 29% | 27% | 26% | 17% | 1% | |  |
|  | Headache | 15% | 83% | 44% | 28% | 24% | 20% | 19% | 18% | 16% | 16% | 10% | 1% | |  |
|  | General weakness | 24% | 76% | 59% | 42% | 37% | 28% | 26% | 26% | 23% | 23% | 13% | 1% | |  |
|  | Myalgia | 35% | 64% | 38% | 27% | 24% | 20% | 19% | 18% | 17% | 16% | 12% | 0% | |  |
|  | Poor sleep quality | 60% | 38% | 30% | 23% | 21% | 19% | 18% | 18% | 17% | 17% | 11% | 1% | |  |
|  | Handwriting deficit | 62% | 36% | 22% | 14% | 11% | 9% | 8% | 8% | 7% | 7% | 3% | 2% | |  |
|  | Excessive sweating | 69% | 29% | 21% | 19% | 17% | 15% | 15% | 15% | 14% | 14% | 8% | 2% | |  |
| Neurological symptoms/sequelae | |  | <2 | 2 | 4 | 6 | 8 | 10 | 12 | 14 | 16 | ≥18 |  | |  |
|  | Impaired balance | 20% | 80% | 46% | 30% | 26% | 21% | 19% | 19% | 18% | 17% | 12% | 0% | |  |
|  | Concentration deficit | 26% | 74% | 46% | 36% | 31% | 25% | 24% | 24% | 22% | 22% | 13% | 0% | |  |
|  | Memory deficit | 49% | 49% | 35% | 27% | 23% | 20% | 19% | 19% | 18% | 17% | 11% | 2% | |  |
|  | Impaired coordination (ataxia) | 54% | 44% | 25% | 17% | 16% | 11% | 10% | 10% | 9% | 9% | 6% | 1% | |  |
|  | Dysphasia (speaking) | 56% | 43% | 21% | 15% | 13% | 11% | 10% | 10% | 8% | 8% | 5% | 1% | |  |
|  | Tremor | 62% | 38% | 21% | 17% | 16% | 13% | 13% | 12% | 11% | 11% | 7% | 0% | |  |
|  | Impaired consciousness | 64% | 35% | 16% | 10% | 9% | 7% | 6% | 6% | 5% | 5% | 3% | 1% | |  |
|  | Sensory impairment | 71% | 28% | 20% | 15% | 15% | 13% | 13% | 13% | 11% | 11% | 8% | 1% | |  |
|  | Pareses | 83% | 17% | 11% | 8% | 8% | 7% | 7% | 7% | 6% | 6% | 4% | 0% | |  |
|  | Hearing impairment | 84% | 16% | 12% | 11% | 10% | 9% | 9% | 9% | 9% | 9% | 6% | 0% | |  |
|  | Dysphagia (swallowing) | 90% | 9% | 5% | 3% | 3% | 2% | 2% | 2% | 2% | 1% | 1% | 0% | |  |
|  | Seizures | 92% | 7% | 4% | 3% | 3% | 3% | 3% | 3% | 3% | 3% | 2% | 1% | |  |

**Appendix 4**. Presence of post-encephalitic syndrome (PES) reported by adults and children at 6, 12, and 18 months after TBE symptom onset

Mild PES: presence of 2–3 subjective symptoms reported due to TBE virus infection, following the definition in (3). Moderate PES: 4–5 subjective symptoms. Severe PES: ≥6 subjective symptoms.

**Appendix 5**. Factors associated with time to recovery from TBE: Results of univariable logistic regression analyses

| **Exposure of Interest** | **Levels** | **Cases** | **Recovered cases** | **Median time to recovery (months)** | **HR** | **95% CI** | **AIC** |
| --- | --- | --- | --- | --- | --- | --- | --- |
| Age group at onset | <18 years | 59 | 56 (95%) | 1.8 | 1.79 | 1.25 – 2.56 | 4057.78 |
|  | 18–39 years | 80 | 65 (81%) | 3.0 | Ref. |  |  |
|  | 40–49 years | 80 | 49 (61%) | 10.5 | 0.55 | 0.38 – 0.80 |  |
|  | ≥50 years | 304 | 182 (60%) | 7.5 | 0.56 | 0.42 – 0.75 |  |
| Sex | Male | 333 | 227 (68%) | 5.0 | Ref. |  | 4108.09 |
|  | Female | 190 | 125 (66%) | 6.0 | 0.88 | 0.71 – 1.09 |  |
| Acute severity | Mild | 96 | 82 (85%) | 2.0 | Ref. |  | 4063.89 |
|  | Moderate | 320 | 222 (69%) | 6.0 | 0.55 | 0.43 – 0.71 |  |
|  | Severe | 107 | 48 (45%) | * | 0.29 | 0.20 – 0.42 |  |
| Comorbidity at onset | Absent | 298 | 224 (75%) | 5.0 | Ref. |  | 4091.34 |
|  | Present | 225 | 128 (57%) | 9.0 | 0.63 | 0.51 – 0.78 |  |
| Hypertension at onset | Absent | 411 | 293 (71%) | 6.0 | Ref. |  | 4096.09 |
|  | Present | 112 | 59 (53%) | 12.0 | 0.61 | 0.46 – 0.81 |  |

* Only 45% of cases in this stratum had recovered by 18 months, hence the median time to recovery could not be determined.

HR = hazard ratio; CI = confidence interval; AIC = Akaike information criterion; Ref. = reference

**Appendix 6**. Definition of acute TBE disease severity for the 581 TBE cases who participated at the first data collection point. Source: (4)


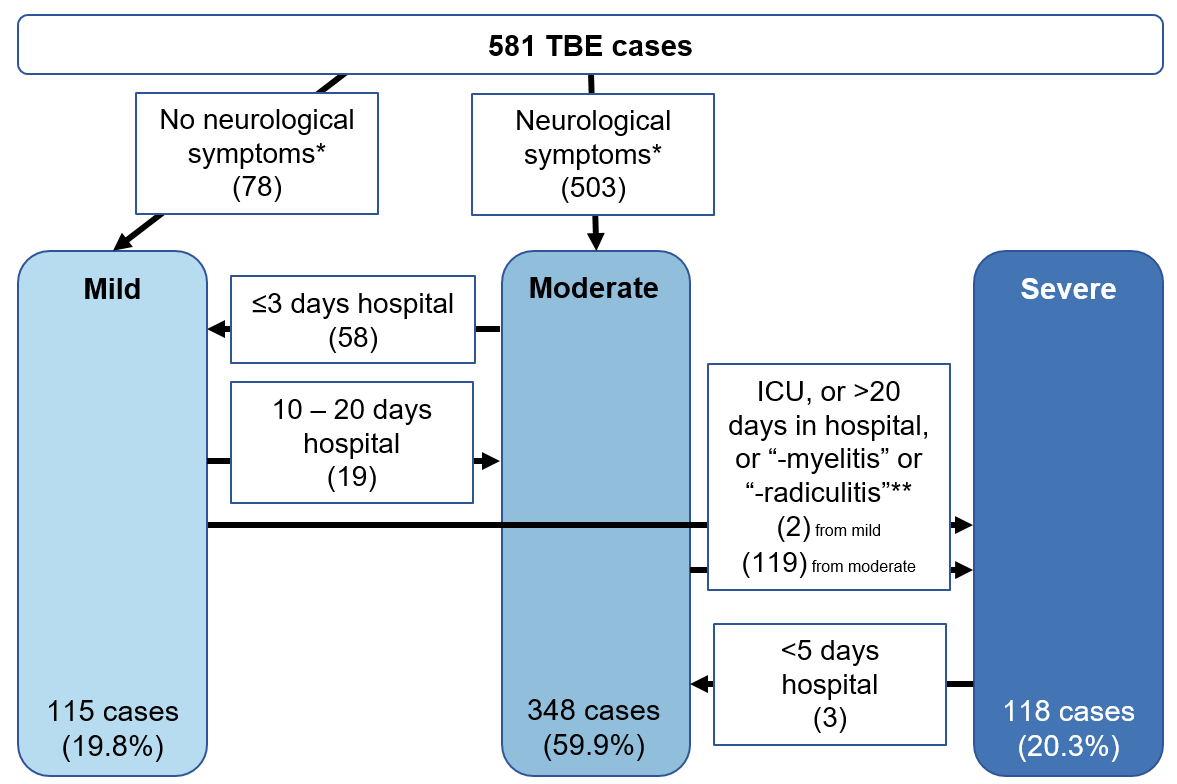


ICU: intensive care unit

* symptoms occurring at any point during the acute disease course. At least 1 of: ataxia, sensory impairment, dysphasia, dysphagia, pareses, impaired consciousness, seizures, hearing impairment, respiratory difficulty/paralysis

** according to hospital discharge summaries

**References**

1. Bruno A, Akinwuntan AE, Lin C, Close B, Davis K, Baute V, et al. Simplified modified rankin scale questionnaire: reproducibility over the telephone and validation with quality of life. Stroke. 2011;42(8):2276-9.

2. Textor J, van der Zander B, Gilthorpe MS, Liskiewicz M, Ellison GT. Robust causal inference using directed acyclic graphs: the R package 'dagitty'. Int J Epidemiol. 2016;45(6):1887-94.

3. Bogovič P, Stupica D, Rojko T, Lotrič-Furlan S, Avšič-Županc T, Kastrin A, et al. The long-term outcome of tick-borne encephalitis in Central Europe. Ticks Tick Borne Dis. 2018;9(2):369-78.

4. Nygren TM, Pilic A, Böhmer MM, Wagner-Wiening C, Went S-B, Wichmann O, et al. Tick-Borne Encephalitis: Acute Clinical Manifestations and Severity in 581 cases from Germany, 2018-2020. J Infect. 2023.
